# Stress-Induced Changes in the Expression of the Clock Protein PERIOD1 in the Rat Limbic Forebrain and Hypothalamus: Role of Stress Type, Time of Day, and Predictability

**DOI:** 10.1371/journal.pone.0111166

**Published:** 2014-10-22

**Authors:** Sherin Al-Safadi, Aya Al-Safadi, Marie Branchaud, Spencer Rutherford, Arun Dayanandan, Barry Robinson, Shimon Amir

**Affiliations:** 1 Department of Biology, Concordia University, Montréal, Quebéc, Canada; 2 Department of Psychology, Center for Studies in Behavioral Neurobiology, Concordia University, Montréal, Quebéc, Canada; University of Texas Southwestern Medical Center, United States of America

## Abstract

Stressful events can disrupt circadian rhythms in mammals but mechanisms underlying this disruption remain largely unknown. One hypothesis is that stress alters circadian protein expression in the forebrain, leading to functional dysregulation of the brain circadian network and consequent disruption of circadian physiological and behavioral rhythms. Here we characterized the effects of several different stressors on the expression of the core clock protein, PER1 and the activity marker, FOS in select forebrain and hypothalamic nuclei in rats. We found that acute exposure to processive stressors, restraint and forced swim, elevated PER1 and FOS expression in the paraventricular and dorsomedial hypothalamic nuclei and piriform cortex but suppressed PER1 and FOS levels exclusively in the central nucleus of the amygdala (CEAl) and oval nucleus of the bed nucleus of the stria terminalis (BNSTov). Conversely, systemic stressors, interleukin-1β and 2-Deoxy-D-glucose, increased PER1 and FOS levels in all regions studied, including the CEAl and BNSTov. PER1 levels in the suprachiasmatic nucleus (SCN), the master pacemaker, were unaffected by any of the stress manipulations. The effect of stress on PER1 and FOS was modulated by time of day and, in the case of daily restraint, by predictability. These results demonstrate that the expression of PER1 in the forebrain is modulated by stress, consistent with the hypothesis that PER1 serves as a link between stress and the brain circadian network. Furthermore, the results show that the mechanisms that control PER1 and FOS expression in CEAl and BNSTov are uniquely sensitive to differences in the type of stressor. Finally, the finding that the effect of stress on PER1 parallels its effect on FOS supports the idea that *Per1* functions as an immediate-early gene. Our observations point to a novel role for PER1 as a key player in the interface between stress and circadian rhythms.

## Introduction

There is considerable evidence that stress can disrupt circadian behavioral and physiological rhythms in mammals, including humans, but the underlying mechanisms are poorly understood. Although it has been shown that the master circadian clock of the brain, the suprachiasmatic nucleus (SCN), which governs most circadian behavioral and physiological rhythms, is not directly affected by acute stress [1]–[4] the effect of stress on subordinate circadian clocks elsewhere in the brain has not been systematically investigated.

Circadian clock proteins, which form the core of the mammalian circadian oscillator [5]–[7], are expressed rhythmically in multiple brain regions outside the SCN, including forebrain and hypothalamic structures that play important roles in stress, motivation and emotion regulation [6], [8]–[11]. It has been previously shown that the expression of clock proteins in many of these brain regions can be altered by manipulations that affect energy balance, such as restricted feeding, by treatment with drugs of abuse, and by various hormonal manipulations that affect behavioral state, including changes in circulating levels of the adrenal glucocorticoid stress hormone, corticosterone (CORT) [12]–[19].

One candidate protein that may mediate the effect of stress on the circadian system is the core clock protein PER1. As shown previously, PER1 expression is induced by predator scent, physical and inflammatory stressors, and plays a role in the regulation of anxiety-related behavior in mammals [4], [20]–[24]. Furthermore, PER1 is rapidly induced by stress in peripheral organs through a glucocorticoid response element (GRE) on the promoter region of its gene [25]. Notably, *Per1* has been implicated in behavioral processes such as cocaine sensitization [26], [27] and alcohol drinking behavior in mice [28], demonstrating that it can also play a role outside of the clock machinery.

To identify extra-SCN nodes of interaction between the circadian and stress systems, we investigated the effect of stress on PER1 expression in select limbic forebrain and hypothalamic structures, including the oval nucleus of the bed nucleus of the stria terminalis (BNSTov), the lateral division of the central nucleus of the amygdala (CEAl), the paraventricular nucleus (PVN), the dorsomedial hypothalamus (DMH), and piriform cortex (Pi). We also examined any changes in stress-induced PER1 expression in the SCN to validate that the master clock remains immune to the effects of acute stress. Our main objective was to study the effects of several qualitatively different stressors, categorized as either processive or systemic [29], on PER1 expression. Processive stressors, such as restraint and forced swim, are defined as noninvasive challenges that elicit acute emotional distress and activate the HPA axis through corticolimbic pathways. Systemic stressors, such as interleukin-1beta (IL-1ß) and 2-Deoxy-D-glucose (2DG) treatments, represent direct physical threats that disrupt internal homeostatic mechanisms, but do not produce an acute affective response [30]–[32]. Because stressors exert their effects contingent upon the time of day of exposure [33], [34], we also investigated the effect of daytime versus nighttime stress on PER1 levels. Furthermore, because the mode of presentation of a stressor plays a major role in determining stress outcome [35]–[37], we studied the effect of repeated daily predictable versus unpredictable stress, where predictability pertains to the repeated administration of the stressor at the same time each day. Lastly, in all experiments, we compared the expression of PER1 to that of the inducible cellular activity marker, FOS to examine the hypothesis that *Per1* might operate as an immediate-early gene (IEG) in the brain [25], [38].

Our results established that stress-induced changes in PER1 expression are strongly subject to influence by the category of stress, the brain region studied, the time of day of stressor administration, and the mode of stressor presentation. Stress-induced alterations in PER1 levels mirrored those of FOS, implicating *Per1* as an IEG. Together, the results support the idea that PER1 may serve as a molecular link between the circadian and stress systems in mammals.

## Materials and Methods

### Animals and Housing

All experimental procedures followed the guidelines of the Canadian Council on Animal Care and were approved by the Animal Care Committee of Concordia University. Adult male Wistar rats (125–150 g) were purchased from Charles River Canada (St. Constant, QC, Canada). The rats were individually housed in clear plastic cages (24 cm wide ×20.5 cm height ×40 cm deep), with free access to food and water, and kept under a 12 h∶12 h light (∼100 lux at cage bottom)/dark schedule (LD) for approximately 2 weeks until they were fully entrained. The cages were equipped with running wheels and were housed in ventilated, light- and sound-attenuated isolation boxes (45 cm wide ×70 cm height ×70 cm deep). Ambient temperature in the isolation chambers was kept at ∼22°C. Running-wheel data were collected by VitalView software (Mini-Mitter, Sunriver, OR, USA). Actograms were acquired and analyzed using Actiview Biological Rhythm Analysis software (Mini-Mitter). The actograms were used to ensure that all rats were stably entrained to the LD cycle.

### Stressors

#### Processive

Rats were exposed to one of two stressors, restraint, consisting of 30 min in custom-made ventilated Plexiglas tubes (7 mm thick, internal diameter of 75 mm, adjustable in length from 130–180 mm), or forced swim (FS) for 15 min in 22°C water in a 40 cm-high and 20 cm-wide Plexiglas tube. Control rats were handled only while receiving tail clips for blood sampling prior to perfusion.

#### Systemic

Rats were exposed to an immune challenge, an i.p. injection of 5 µg/kg human IL-1ß (IL-1ß, Cell Guidance Systems, Carlsbad, CA, USA) in sterile water, or a metabolic challenge, s.c. injection of 250 mg/kg 2-deoxyglucose (2DG, Sigma-Aldrich, Oakville, ON, Canada) in 0.9% saline. Control rats were injected with vehicle only. Rats were tail clipped for blood sampling prior to perfusion.

### Plasma CORT Collection and Analysis

Rats were wrapped in a towel and tail-clipped with a razor for rapid blood collection using capillary tubes (0.5 ml). Samples were centrifuged at 4°C, 13,000 r.p.m. for 10 min, and the plasma was extracted and stored at −80°C. Plasma CORT levels were assessed in duplicates using a CORT Enzyme Immunoassay kit (Enzo Life sciences, Farmingdale, NY, USA) according to the manufacturer's protocol.

### Tissue Preparation & Immunohistochemistry

Rats were deeply anesthetized with sodium pentobarbital (100 mg/kg, i.p.) and perfused transcardially with 300 ml of cold 0.9% saline (4°C, 0.9% NaCl in distilled water), followed by 300 ml of cold 4% paraformaldehyde (4°C, 4% paraformaldehyde in 0.1 M phosphate buffer). Brains were removed and post-fixed for 24 h in 4% paraformaldehyde and stored at 4°C overnight. They were then sliced in 50 µm serial coronal sections on a vibratome, and stored at −20°C in Watson's Cryoprotectant. Immunohistochemistry for PER1 was performed as previously described [15] using an affinity-purified rabbit polyclonal antibody, raised against PER1 (1 24,000 - R1177, EMD-Millipore). Brain sections were incubated (40 h, 4°C) in a primary solution with PER1 polyclonal rabbit antibodies, 2% normal goat serum, 5% milk buffer in a Triton Trizma-buffered saline solution (0.3% Triton, 50 mM Trizma buffer, 0.9% saline). The sections were then incubated in a secondary solution, composed of biotinylated anti-rabbit IgG, raised in goat (1 200, Vector Laboratories, Burlington, ON, Canada). Lastly, the sections were incubated in a tertiary Avidin-Biotin-Peroxidase solution (Vectastain Elite ABC Kit, Vector Laboratories). All sections were rinsed in a 0.5% 3,3-diaminobenzidine (DAB) solution. Immunoreactive (IR) cells were stained using a 0.5% DAB, 0.01% H_2_O_2_ and 8% NiCl_2_ solution. Immunohistochemistry for FOS was performed on a second set of brain sections collected from each rat as previously described [39], using a polyclonal FOS antibody raised in rabbit (1 100,000 - Calbiochem, Gibbstown, NJ, USA).

### Microscopy & Data Analysis

Stained sections were mounted onto gel-coated slides and dehydrated in a series of alcohols and Citrisolv (Fisher), then coverslipped. The sections were examined under a light microscope (Leica, DMR) and identified using a Swanson rat brain atlas [40]. Images of the SCN, PVN, DMH, Pi, BNSTov and CEA were captured using a Sony XC-77 video camera, Scion LG-3 frame grabber (Scion Corporation, Frederick, MD, USA), and Image SXM software (S.D. Barret, v1.95, http://www.ImageSXM.org.uk). The mean number of PER1 and FOS IR cells per region was then calculated for each animal from the counts of six unilateral images showing the highest number of labeled nuclei, as previously described [9], [10]. All images were sampled from the entire nucleus in question, at different rostral-caudal levels. Image capture and cell counts were performed blind. Data were analyzed using a two-way analysis of variance (ANOVA) and a Bonferroni post-hoc test. Differences between groups for the time of day effect of stress were revealed using a one-way ANOVA and Dunnett's post-hoc analysis.

### Experimental Protocol

#### Qualitatively different stressors (processive versus systemic)

Groups of rats (*n* = 4 per group) were acutely exposed to either processive (restraint or FS), or systemic (IL-1ß or 2DG) stressors at ZT2 (ZT0 denotes the onset of the light phase), a time of day when basal levels of CORT are at a nadir. Levels of PER1, FOS and CORT were analyzed 1, 3 and 6 h post-stress onset, corresponding to ZT3, 5 and 8. These time points were selected in order to reflect acute changes in protein levels in the short, medium and long-term, respectively.

#### Time of day of exposure (night versus day stress)

PER1 and FOS expression and CORT levels oscillate over the 24-h period, with levels varying contingent upon the time of day [41]–[43]. To assess the effect of nighttime exposure to stress on changes in PER1 and FOS expression, restraint or 2DG were administered at ZT14, 2 h after the onset of the dark phase and rats (n = 4 per group) were killed 1 h later at ZT15. All nighttime procedures were carried-out under dim red light (∼2 lux) using a Philips 7 W red incandescent light source. Note, in this and subsequent experiments, brains and blood were collected only in the short term, 1 h post-stress onset, as this was the time point when stress had its maximal effect on these measures.

#### Mode of presentation (repeated daily predictable versus unpredictable stress)

Rats (*n* = 4 per group) were assigned to one of four groups: predictable day, predictable night, unpredictable day and unpredictable night. Predictable paradigms entailed exposure to the stressor at the same time each day. Predictable day rats were exposed to restraint stress (30 min) at ZT2 once a day, for 10 days, and were killed at ZT3 on Day 10. Predictable night rats were restrained once a day at ZT14, and were killed at ZT15 on Day 10. Unpredictable day rats were restrained once a day for 10 days at a randomly selected ZT during the day using an online random number generator, except on Day 10, when they were restrained at ZT2 then killed at ZT3. This was done in order to control for the effect of time of day on levels of PER1, FOS and CORT. Unpredictable night rats were restrained once a day at a randomly selected ZT during the night, except for Day 10, when they were restrained at ZT14 and killed at ZT15. All nighttime procedures were carried-out under dim red light (∼2 lux) using a Philips 7 W red incandescent light source.

## Results

### Acute processive stressors increase PER1 and FOS expression in PVN, DMH and Pi, but decreases expression in BNSTov and CEAl

**Figure 1:**
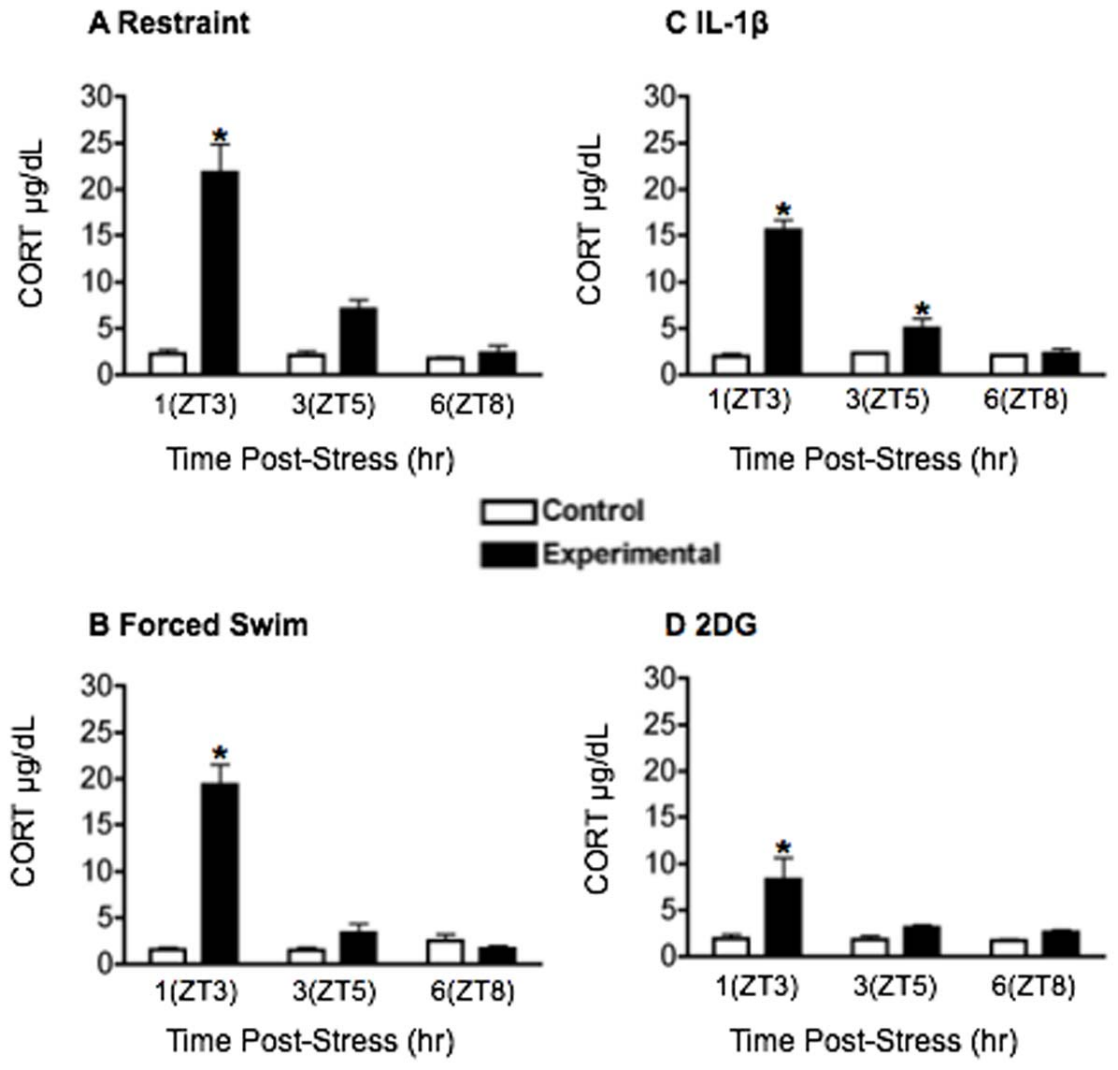
The effect of stress on plasma corticosterone (CORT). Plasma CORT levels in control and stressed (experimental) rats following **A**) 30 min restraint **B**) 15 min forced swim **C**) treatment with 5 µg/kg IL-1β **D**) treatment with 250 mg/kg 2DG. Means ± SEM are shown, *n* = 4 per group; * significant difference from corresponding control group, *p*<0.05.

**Figure 2:**
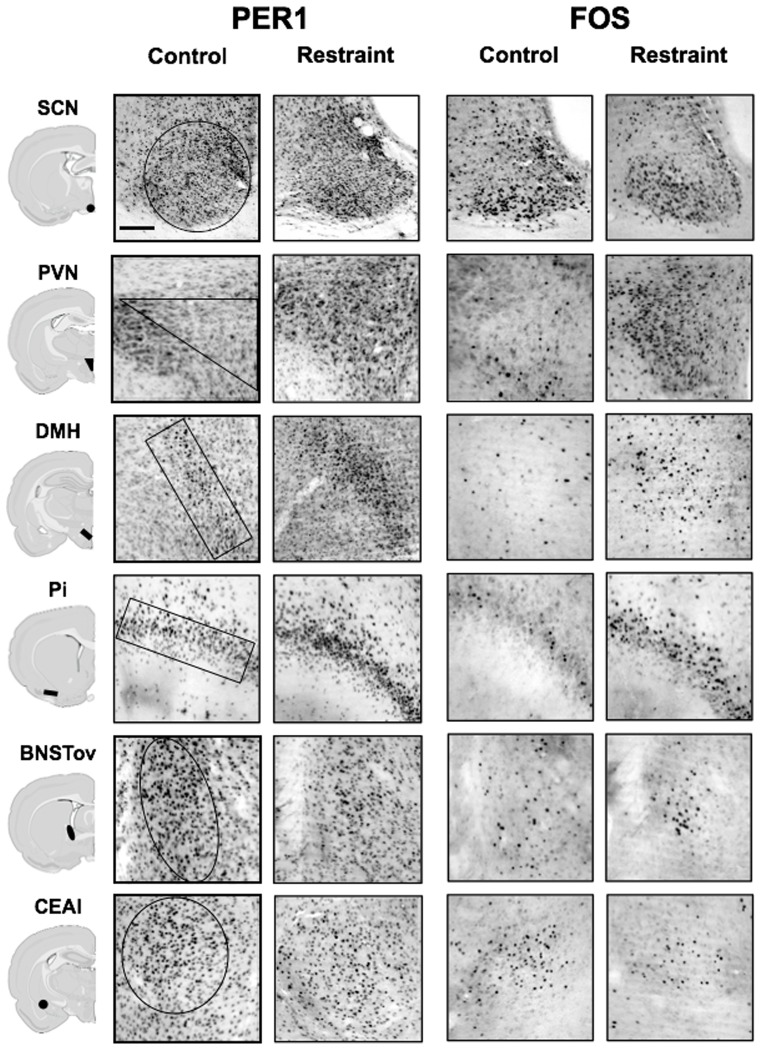
Photomicrographs showing examples of the effect of stress on PER1 and FOS expression in different hypothalamic and forebrain regions. Experimental rats were stressed with a 30 min restraint session at ZT2, and brains collected at ZT3 (scale bar: 100 µm). Schematics of the location of each brain structure under study are shown in the left column.

**Figure 3:**
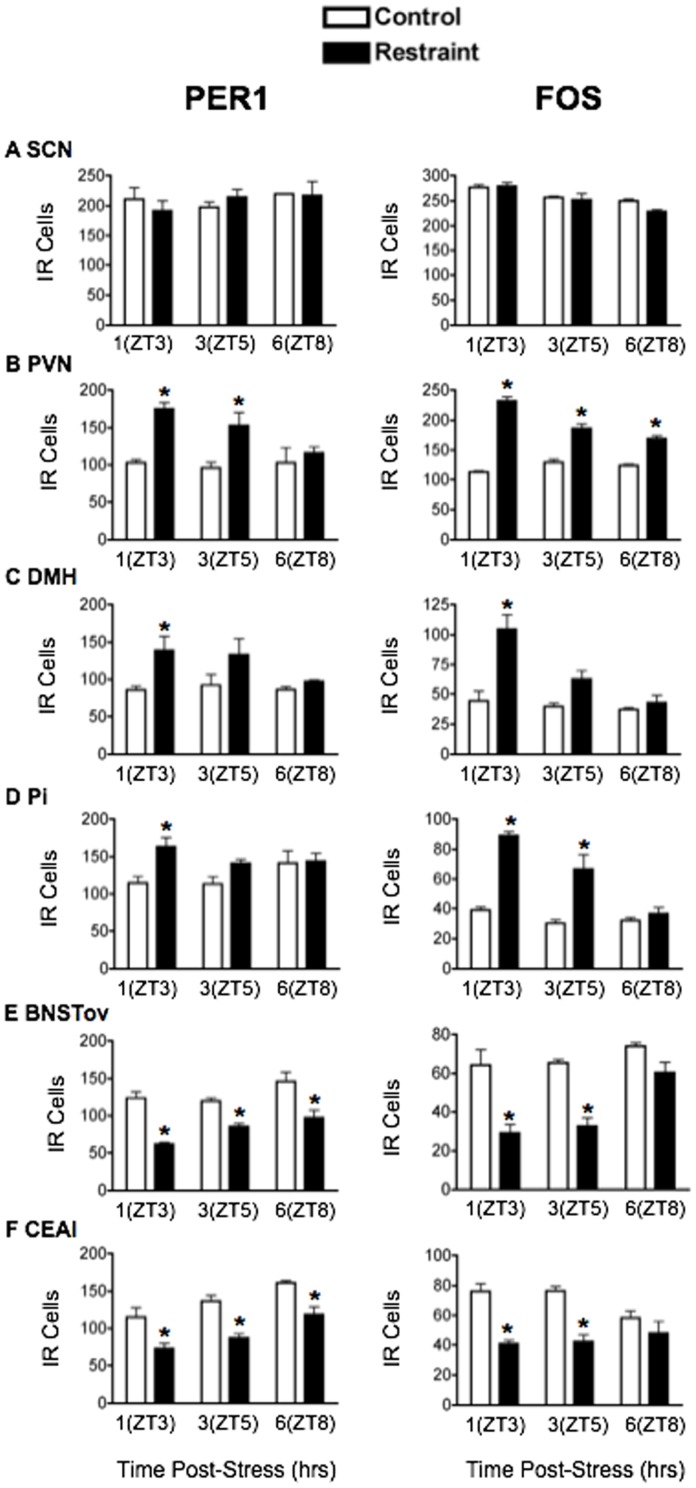
The effect of acute restraint stress on PER1 and FOS expression. Means ± SEM of immunoreactive (IR) cells are shown, *n* = 4 per group; * significant difference from corresponding control group, *p*<0.05.

Restraint: Exposure to a 30-min restraint stressor at ZT2 significantly increased plasma CORT levels (main effect of stress: F_1,12_ = 55.98, *p*<0.001; Fig. 1A). CORT levels peaked 1 h after the onset of the stressor, at ZT3 and returned to baseline 5 h later at ZT8 (main effect of time: F_2,12_ = 28.53, *p*<0.001). The effect of restraint stress on PER1 and FOS expression is shown in Figs. 2 and 3, and statistical analyses in Table 1. PER1 and FOS levels in the SCN were unaffected by restraint at all 3 time points (Fig. 3A), consistent with previous findings [4]. In the PVN, DMH and Pi, the levels of both PER1 and FOS increased significantly after stress, most noticeably 1 h after restraint onset, at ZT3 (Figs. 3B–D). In contrast, restraint induced an opposite effect in the BNSTov and CEAl, where a significant decrease of both PER1 and FOS was observed in the short term, 1 h after stress onset, at ZT3 (Figs. 3E, F). The levels of PER1 and FOS in these two brain regions increased over the next 6 h and reached control levels by ZT8 (Figs. 3E, F).

**Table 1:** Statistical analysis (ANOVA) of the effect of acute restraint stress on PER1 and FOS expression in hypothalamic and limbic brain regions.

Structure/Protein	Restraint	ZT (time of sacrifice)	Restraint × ZT
**SCN/**			
PER1	F_(1,13)_ = 0.01, ns	F_(2,13)_ = 0.44, ns	F_(2,13)_ = 0.5, ns
FOS	F_(1,17)_ = 1.65, ns	F_(2,17)_ = 14.52, *p*<0.001	F_(2,17)_ = 1.49, ns
**PVN/**			
PER	F_(1,15)_ = 23.88, *p*<0.001	F_(2,15)_ = 2.8, ns	F_(2,15)_ = 2.92, ns
FOS	F_(1,17)_ = 245.94, *p*<0.0001	F_(2,17)_ = 9.83, *p*<0.01	F_(2,17)_ = 24.2, *p*<0.0001
**DMH/**			
PER1	F_(1,14)_ = 10.48, *p*<0.01	F_(2,14)_ = 1.54, ns	F_(2,14)_ = 1.25, ns
FOS	F_(1,16)_ = 26.46, *p*<0.0001	F_(2,16)_ = 11.83, *p*<0.0001	F_(2,16)_ = 7.36, *p*<0.01
**Pi/**			
PER1	F_(1,18)_ = 8.11, *p* = 0.01	F_(2,18)_ = 1.01, ns	F_(2,18)_ = 2.12, ns
FOS	F_(1,17)_ = 54.52, *p*<0.0001	F_(2,17)_ = 17.21, *p*<0.0001	F_(2,17)_ = 10.44, *p*<0.01
**BNSTov/**			
PER1	F_(1,16)_ = 54.87, *p*<0.0001	F_(2,16)_ = 6.74, *p*<0.01	F_(2,16)_ = 1.48, ns
FOS	F_(1,17)_ = 42.82, *p*<0.0001	F_(2,17)_ = 9.31, *p*<0.01	F_(2,17)_ = 2.54, ns
**CEAl/**			
PER1	F_(1,17)_ = 35.74, *p*<0.0001	F_(2,17)_ = 12.31, *p*<0.001	F_(2,17)_ = 0.12, ns
FOS	F_(1,16)_ = 40.03, *p*<0.0001	F_(2,16)_ = 0.82, ns	F_(2,16)_ = 3.69, *p*<0.05

ns, not significant.

**Figure 4:**
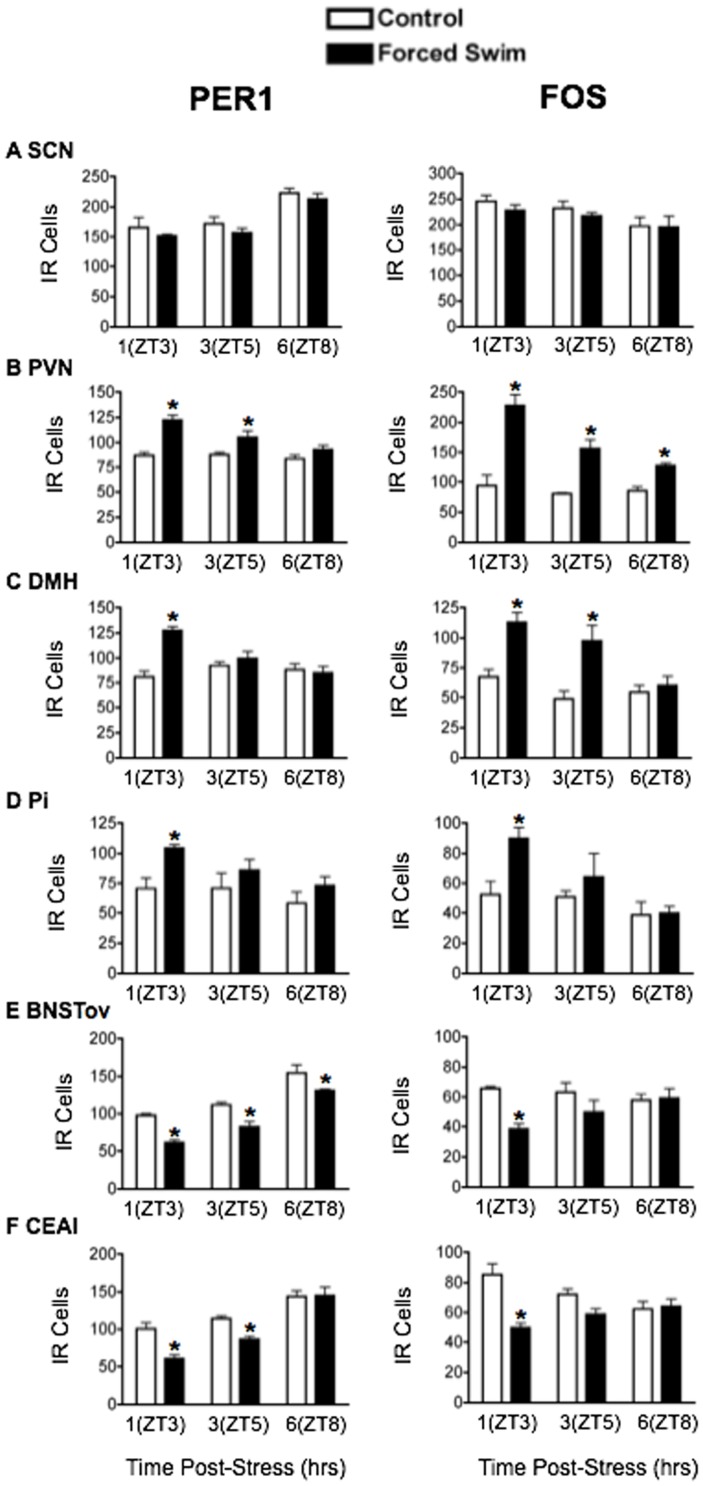
The effect of acute forced swim stress on PER1 and FOS expression. Means ± SEM of immunoreactive (IR) cells are shown, *n* = 4 per group; * significant difference from corresponding control group, *p*<0.05.

Forced Swim: FS significantly elevated plasma CORT levels (main effect of stress: F_1,18_ = 53.83, *p*<0.0001; Fig. 1B). CORT levels peaked 1 h post stress at ZT3 and returned to baseline 5 h later, by ZT8 (main effect of time: F_2,18_ = 40.88, *p*<0.0001). The effect of FS stress on PER1 and FOS expression is shown in Fig. 4, with statistical analyses in Table 2. PER1 and FOS levels in the SCN were unaffected by FS at all time points (Fig. 4A). As was the case with restraint stress, acute exposure to FS significantly increased the expression of PER1 and FOS in the PVN, DMH and Pi. This effect was most pronounced in the short term, 1 h after FS onset, at ZT3, with levels of both PER1 and FOS returning to baseline 6 h later, by ZT8 (Figs. 4B–D). Significantly, as with restraint, exposure to FS stress transiently suppressed PER1 and FOS levels in the BNSTov and CEAl (Figs. 4E, F), pointing to a brain region-specific effect of processive stress on PER1 and FOS expression.

**Table 2:** Statistical analysis (ANOVA) of the effect of acute forced swim stress on PER1 and FOS expression in hypothalamic and limbic brain regions.

Structure/Protein	Forced Swim	ZT (time of sacrifice)	Forced Swim × ZT
**SCN/**			
PER1	F_(1,16)_ = 2.86, ns	F_(2,16)_ = 24.8, *p*<0.0001	F_(2,16)_ = 0.04, ns
FOS	F_(1,18)_ = 0.94, ns	F_(2,18)_ = 4.01, *p*<0.05	F_(2,18)_ = 1.49, ns
**PVN/**			
PER1	F_(1,18)_ = 31.61, *p*<0.0001	F_(2,18)_ = 6.5, *p*<0.01	F_(2,18)_ = 4.36, *p*<0.05
FOS	F_(1,17)_ = 245.94, *p*<0.0001	F_(2,17)_ = 9.83, *p*<0.01	F_(2,17)_ = 24.2, *p*<0.0001
**DMH/**			
PER1	F_(1,17)_ = 12.49, *p*<0.01	F_(2,17)_ = 4.27, *p*<0.05	F_(2,17)_ = 10.04, *p*<0.01
FOS	F_(1,16)_ = 25.47, *p* = 0.0001	F_(2,16)_ = 8.4, *p*<0.001	F_(2,16)_ = 4.53, *p*<0.05
**Pi/**			
PER1	F_(1,16)_ = 8.78, *p*<0.01	F_(2,16)_ = 3.4, ns	F_(2,16)_ = 0.8, ns
FOS	F_(1,17)_ = 6.4, *p*<0.05	F_(2,17)_ = 7.6, *p*<0.01	F_(2,17)_ = 2.49, ns
**BNSTov/**			
PER1	F_(1,18)_ = 37.6, *p*<0.0001	F_(2,18)_ = 60.81, *p*<0.0001	F_(2,18)_ = 0.65, ns
FOS	F_(1,15)_ = 8.66, *p* = 0.01	F_(2,15)_ = 0.74, ns	F_(2,15)_ = 3.66, ns
**CEAl/**			
PER1	F_(1,18)_ = 13.79, *p*<0.01	F_(2,18)_ = 39.24, *p*<0.001	F_(2,18)_ = 4.0, *p*<0.05
FOS	F_(1,18)_ = 15.25, *p* = 0.001	F_(2,18)_ = 0.35, ns	F_(2,18)_ = 7.39, *p*<0.01

ns, not significant.

### Acute systemic stressors increase PER1 and FOS expression in PVN, DMH, Pi, BNSTov and CEAl

**Figure 5:**
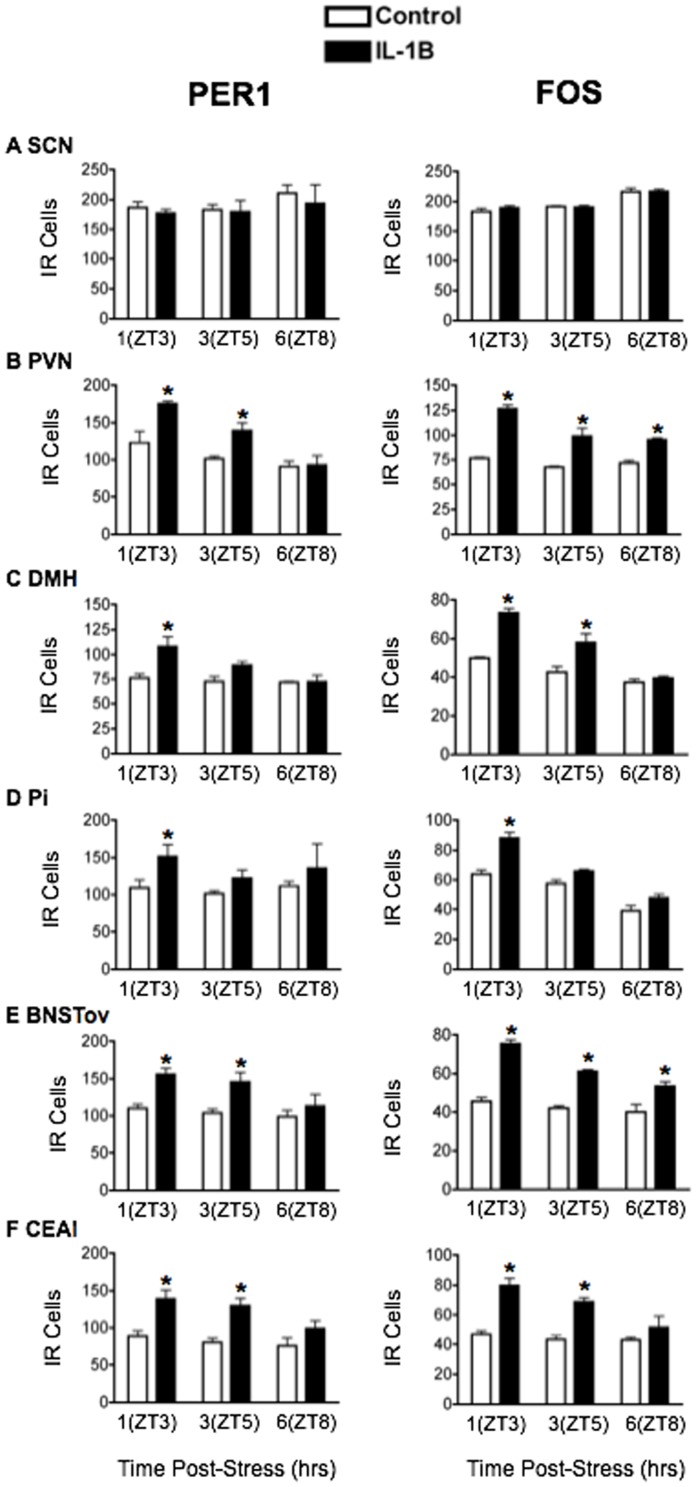
The effect of acute IL-1β (5 µg/kg) treatment on PER1 and FOS expression. Means ± SEM of immunoreactive (IR) cells are shown, *n* = 4 per group; * significant difference from corresponding control group, *p*<0.05.

IL-1β: Plasma CORT levels were significantly elevated 1 h following exposure to IL-1β (main effect of stress: F_1,18_ = 100.77, *p*<0.0001; Fig. 1C) and returned to control levels by ZT8 (main effect of time: F_2,18_ = 53.14, *p*<0.0001). Fig. 5 shows the effects of IL-1β on PER1 and FOS levels, with corresponding statistical analyses in Table 3. IL-1β had no effect on PER1 and FOS levels in the SCN (Fig. 5A). Furthermore, as with restraint and FS stress, acute treatment with IL-1β significantly elevated PER1 and FOS in the PVN, DMH and Pi (Figs. 5B–D). In contrast to processive stressors, however, acute exposure to IL-1β also significantly increased the expression of PER1 and FOS in the BNSTov and CEAl (Figs. 5E, F).

**Table 3:** Statistical analysis (ANOVA) of the effect of acute IL-1β challenge on PER1 and FOS expression in hypothalamic and limbic brain regions.

Structure/Protein	IL-1β	ZT (time of sacrifice)	IL-1β × ZT
**SCN/**			
PER1	F_(1,16)_ = 0.59, ns	F_(2,16)_ = 1.09, ns	F_(2,16)_ = 0.09, ns
FOS	F_(1,16)_ = 0.21, ns	F_(2,16)_ = 30.06, *p*<0.001	F_(2,16)_ = 0.34, ns
**PVN/**			
PER1	F_(1,16)_ = 14.39, *p*<0.01	F_(2,16)_ = 16.03, *p*<0.001	F_(2,16)_ = 3.27, ns
FOS	F_(1,17)_ = 106.23, *p*<0.0001	F_(2,17)_ = 11.97, *p*<0.001	F_(2,17)_ = 4.91, *p*<0.05
**DMH/**			
PER1	F_(1,16)_ = 13.22, *p*<0.01	F_(2,16)_ = 6.77, *p*<0.01	F_(2,16)_ = 4.33, *p*<0.05
FOS	F_(1,16)_ = 34.80, *p*<0.0001	F_(2,16)_ = 32.25, *p*<0.0001	F_(2,16)_ = 7.19, *p*<0.01
**Pi/**			
PER1	F_(1,18)_ = 4.64, *p*<0.05	F_(2,18)_ = 0.62, ns	F_(2,18)_ = 0.23, ns
FOS	F_(1,16)_ = 31.85, *p*<0.001	F_(2,16)_ = 60.62, *p*<0.001	F_(2,16)_ = 4.63, *p*<0.05
**BNSTov/**			
PER1	F_(1,16)_ = 14.29, *p*<0.01	F_(2,16)_ = 2.93, ns	F_(2,16)_ = 1.17, ns
FOS	F_(1,16)_ = 137.24, *p*<0.0001	F_(2,16)_ = 19.71, *p*<0.0001	F_(2,16)_ = 6.82, *p*<0.01
**CEAl/**			
PER1	F_(1,17)_ = 26.79, *p*<0.01	F_(2,17)_ = 3.74, *p*<0.05	F_(2,17)_ = 1.32, ns
FOS	F_(1,17)_ = 42.43, *p*<0.0001	F_(2,17)_ = 7.26, *p*<0.01	F_(2,17)_ = 4.42, *p*<0.05

ns, not significant.

**Figure 6:**
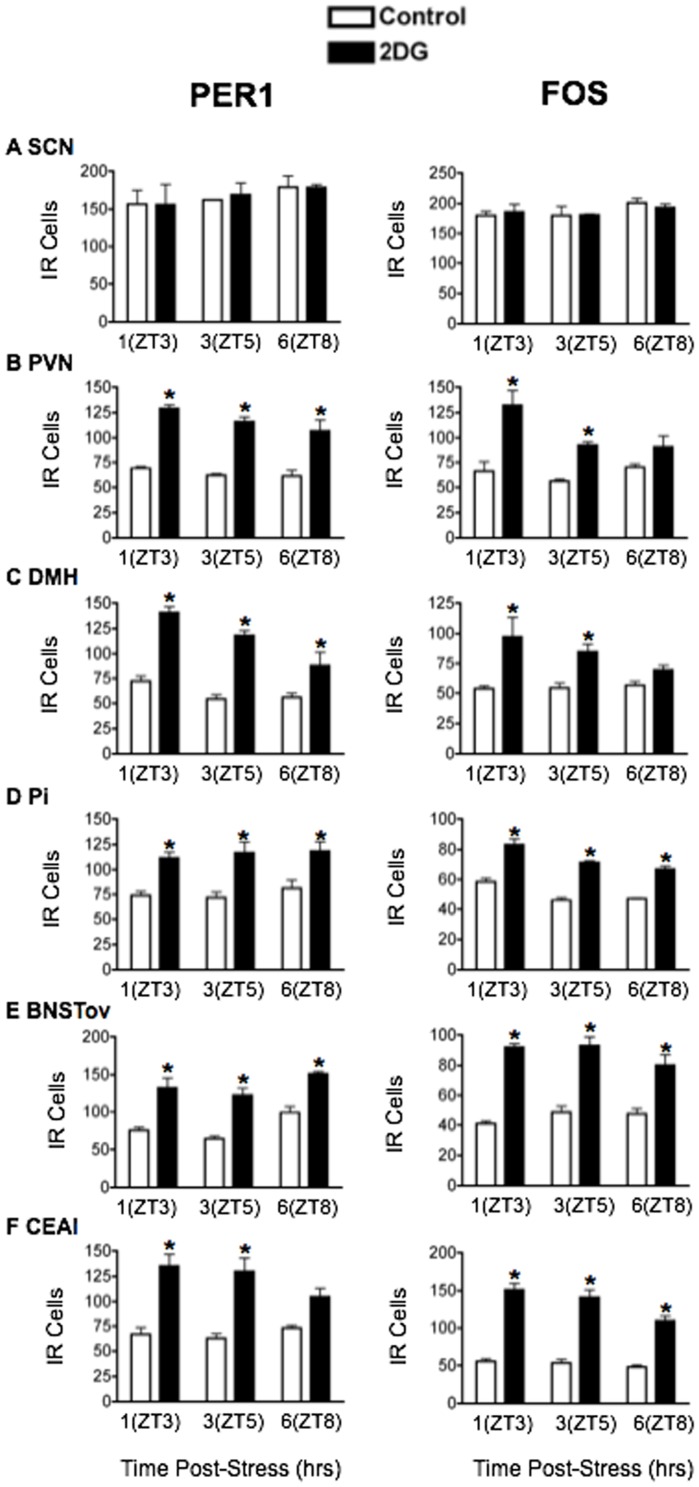
The effect of acute 2DG (250 mg/kg) treatment on PER1 and FOS expression. Means ± SEM of immunoreactive (IR) cells are shown, *n* = 4 per group; * significant difference from corresponding control group, *p*<0.05.

2DG: Treatment with 2DG significantly elevated plasma CORT (main effect of stress: F_1,18_ = 12.36, *p*<0.01; Fig. 1D) with levels peaking in the short term at ZT3 and returning to baseline by ZT8 (main effect of time: F_2,18_ = 5.25, *p*<0.05). The effects of 2DG on PER1 and FOS are shown in Fig. 6 and statistical analyses in Table 4. No changes in PER1 and FOS expression were seen in the SCN following treatment with 2DG (Fig. 6A). Acute exposure to 2DG significantly increased levels of PER1 and FOS in the short term at ZT3 in all other brain regions studied (Figs. 6B–F).

**Table 4:** Statistical analysis (ANOVA) of the effect of acute 2DG challenge on PER1 and FOS expression in hypothalamic and limbic brain regions.

Structure/Protein	2DG	ZT (time of sacrifice)	2DG × ZT
**SCN/**			
PER1	F_(1,15)_ = 0.02, ns	F_(2,15)_ = 0.98, ns	F_(2,15)_ = 0.03, ns
FOS	F_(1,16)_ = 0, ns	F_(2,16)_ = 1.36, ns	F_(2,16)_ = 0.22, ns
**PVN/**			
PER1	F_(1,18)_ = 123.67, *p*<0.0001	F_(2,18)_ = 3.45, ns	F_(2,18)_ = 0.75, ns
FOS	F_(1,18)_ = 33.13, *p*<0.0001	F_(2,18)_ = 4.36, *p*<0.05	F_(2,18)_ = 3.53, ns
**DMH/**			
PER1	F_(1,18)_ = 86.6, *p*<0.0001	F_(2,18)_ = 11.69, *p*<0.001	F_(2,18)_ = 3.79, *p*<0.05
FOS	F_(1,17)_ = 30.25, *p*<0.0001	F_(2,17)_ = 1.78, ns	F_(2,17)_ = 2.76, ns
**Pi/**			
PER1	F_(1,18)_ = 39.3 *p*<0.0001	F_(2,18)_ = 0.44, ns	F_(2,18)_ = 0.16, ns
FOS	F_(1,18)_ = 154.44, *p*<0.0001	F_(2,18)_ = 21.65, *p*<0.0001	F_(2,18)_ = 0.82, ns
**BNSTov/**			
PER1	F_(1,17)_ = 73.73, *p*<0.0001	F_(2,17)_ = 7.91, *p*<0.01	F_(2,17)_ = 0.1, ns
FOS	F_(1,18)_ = 133.18, *p*<0.0001	F_(2,18)_ = 1.23, ns	F_(2,18)_ = 2.16, ns
**CEAl/**			
PER1	F_(1,18)_ = 60.05, *p*<0.0001	F_(2,18)_ = 0.94, ns	F_(2,18)_ = 2.81, ns
FOS	F_(1,18)_ = 220.0, *p*<0.0001	F_(2,18)_ = 7.15, *p*<0.01	F_(2,18)_ = 3.62, *p*<0.05

ns, not significant.

**Figure 7:**
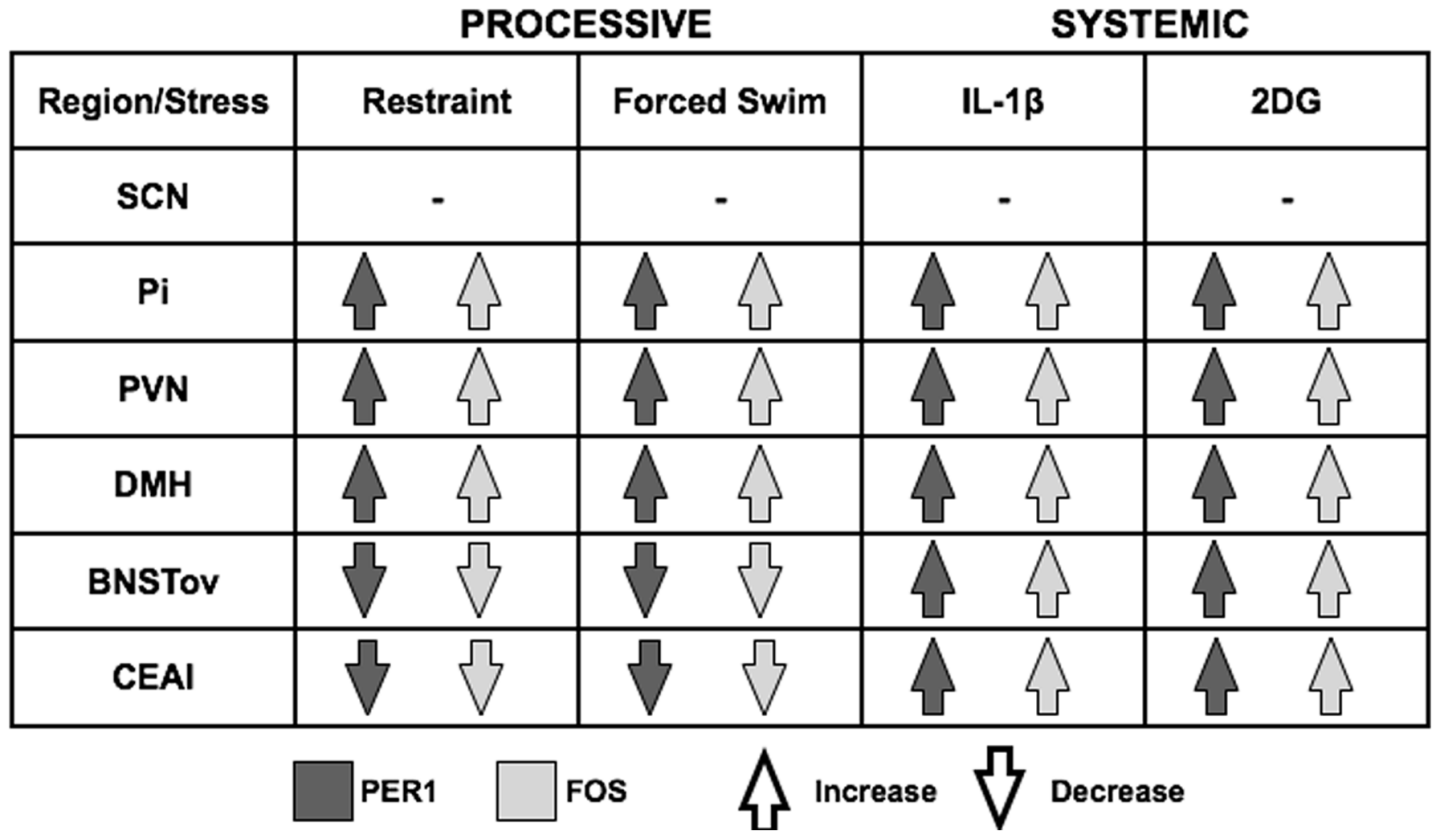
Summary of the effect of acute exposure to categorically-different stressors on PER1 and FOS expression. Note the distinct effects of processive versus systemic stress on PER1 and FOS expression in in the BNSTov and CEAl.

In summary, the results show that acute exposure to stress can rapidly modify PER1 expression in specific hypothalamic and forebrain regions important in stress, motivation and emotion regulation, without affecting expression in the SCN. These stress-induced changes closely mirror those of FOS and they vary as a function of brain region and type of stress, with systemic stressors invariably increasing PER1 and FOS expression in all regions studied, including the PVN, DMH, Pi, BNSTov and CEAl, and processive stressors selectively decreasing expression in the BNSTov and CEAl, while increasing expression in the PVN, DMH and Pi (Fig. 7).

### Nighttime stress-induced changes in PER1 and FOS expression in the limbic forebrain and hypothalamus

Because the effect of stress can be influenced by the time of exposure [44]–[46] and because basal levels of PER1 and FOS expression and of circulating CORT follow a diurnal rhythm [47]–[50], we examined whether acute stress at night would differentially affect PER1 and FOS expression in the short term, compared to daytime stress. PER1, FOS and CORT were analyzed at ZT15, 1 h after the onset of either a processive (restraint) or systemic (2DG) stressor.

**Figure 8:**
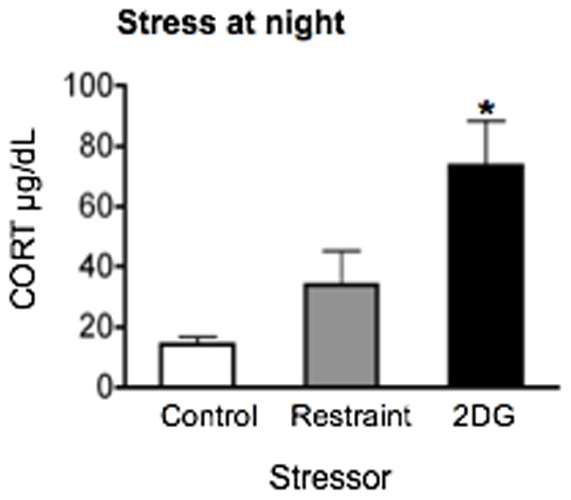
The effect of time of day of stress exposure on plasma corticosterone (CORT). Rats were exposed to 30 min restraint stress or treatment with 250 mg/kg 2DG administered during the nighttime at ZT14. Rats were killed at ZT15, 1 hour after stress onset. Means ± SEM are shown, *n* = 4 per group; * significant difference from corresponding control group, *p*<0.05.

**Figure 9:**
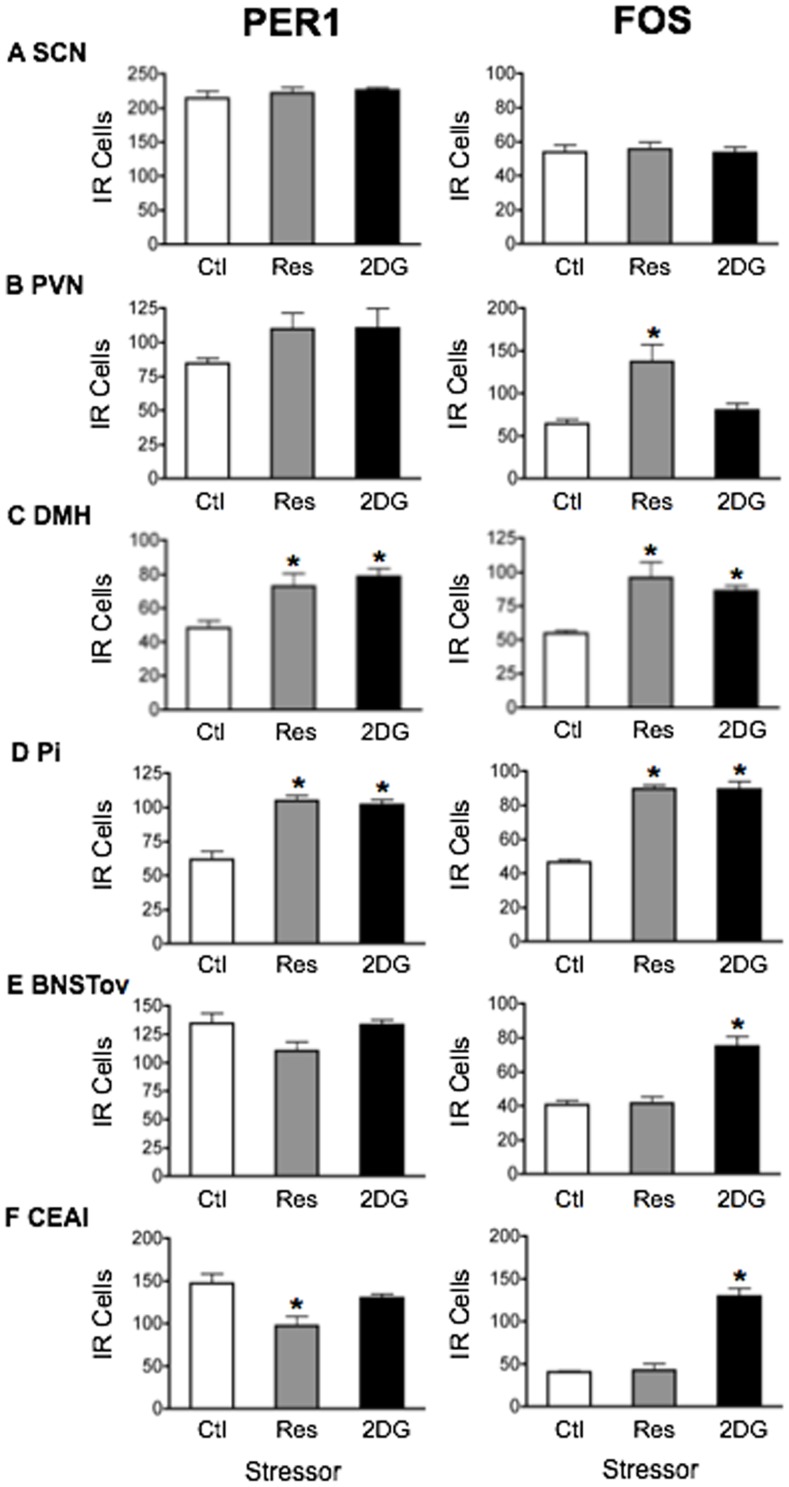
The effect of acute nighttime stress on PER1 and FOS expression. Rats were exposed to 30 min restraint or treatment with 250 mg/kg 2DG, administered during the nighttime at ZT14, and then killed at ZT15. Means ± SEM of immunoreactive (IR) cells are shown, *n* = 4 per group; * significant difference from corresponding control group, *p*<0.05.

Plasma CORT varied across all 3 groups (F_2,9_ = 7.68, *p*<0.05; Fig. 8). CORT levels were significantly higher than control levels following the 2DG challenge (Fig. 8, *p*<0.05), though not after restraint. Neither nighttime restraint nor 2DG treatment had any effect on PER1 or FOS levels in the SCN (Fig. 9A). Similarly, there were no effects of either stressor on PER1 expression in the PVN. FOS levels in the PVN were significantly increased only in response to restraint (F_2,8_ = 8.11, *p*<0.01; Fig. 9B). In the DMH (Fig. 9C) and in the Pi (Fig. 9D), PER1 and FOS expression were significantly increased by both stressors (DMH: PER1, F_2,9_ = 7.38, *p*<0.05; FOS, F_2,9_ = 9.13, *p*<0.01; Pi: PER1, F_2,9_ = 25.93, *p*<0.01; FOS, F_2,9_ = 65.44, *p*<0.0001). Nighttime restraint, though not 2DG, significantly suppressed PER1 levels in the CEAl (F_2,9_ = 7.05, *p*<0.01; Fig. 9F). There was no effect of either stressor on PER1 expression in the BNSTov (Fig. 9E). FOS expression varied significantly across the stress groups in the BNSTov (F_2,9_ = 21.07, *p*<0.01, Fig. 9E) and CEAl (F_2,9_ = 51.57, *p*<0.01; Fig. 9F). Both regions exhibited an increase in FOS expression following exposure to 2DG but not restraint (Figs. 9E, F).

Taken together, the results show that although the changes in PER1 and FOS expression after nighttime stress resemble those seen following daytime stress, daytime stressors induce more robust changes, highlighting the importance of time of day of stress exposure on PER1 and FOS expression in the nuclei studied.

### Effects of repeated daily unpredictable or predicable restraint stress on PER1 and FOS expression

The effect of stress on behavior and physiology is known to depend on factors such as chronicity (acute versus repeated daily stressors) [51] and predictability (predictable versus unpredictable stressors) [52]. To assess the importance of the mode of presentation of a stressor on PER1 and FOS expression, we investigated the effect of daily predictable versus unpredictable restraint stress during the light and dark phase.

**Figure 10:**
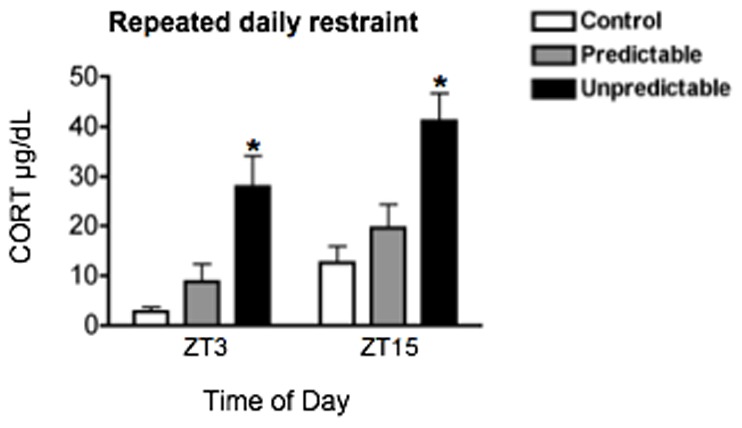
The effect of stress predictability on plasma corticosterone (CORT). Plasma CORT levels in response to 30 min restraint at ZT2 or ZT14 in rats previously exposed to repeated daily predictable vs. unpredictable restraint stress for 10 days during the day or night. Rats were killed 1 h after stress onset, at ZT 3 or ZT15. Means ± SEM are shown, *n* = 4 per group; * significant difference from corresponding control group, *p*<0.05.

**Figure 11:**
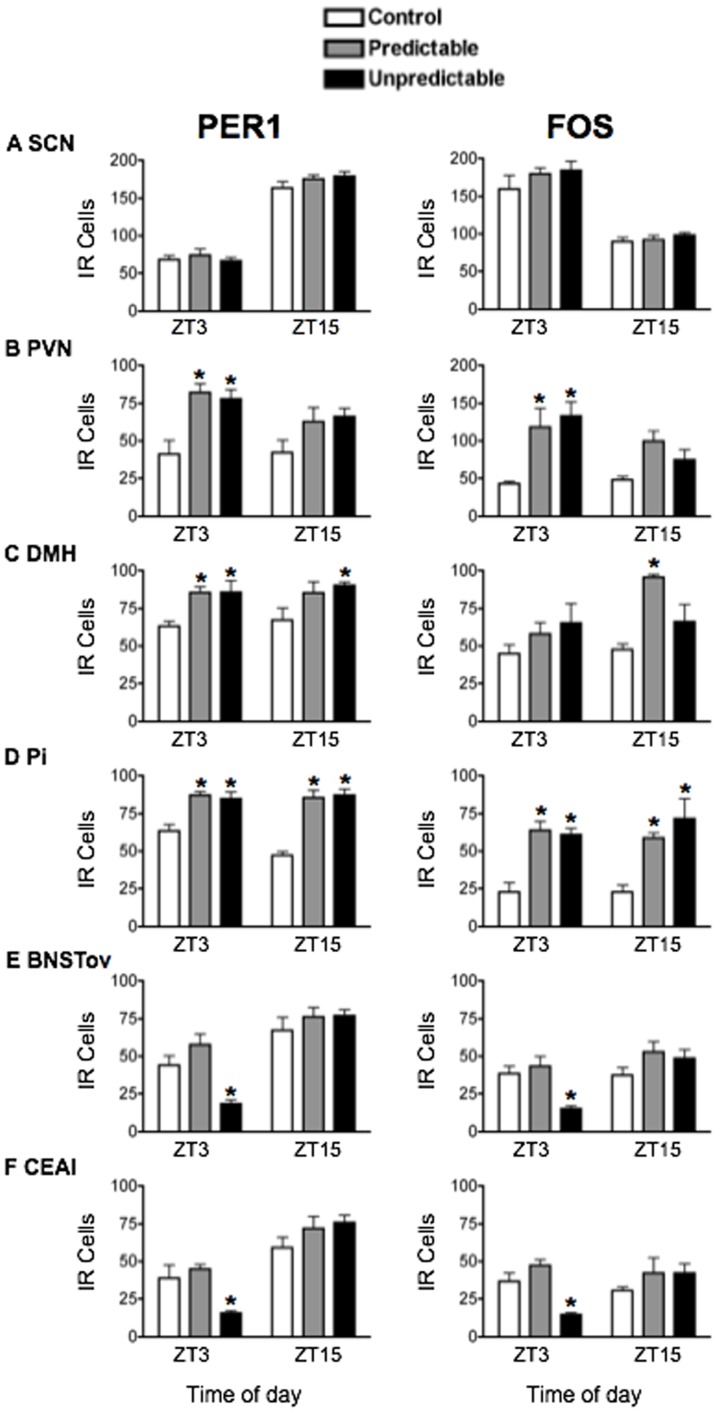
The effect of stress predictability on PER1 and FOS expression. Rats were exposed to repeated daily predictable vs. unpredictable 30 min restraint stress, administered at either ZT2 or ZT14, and killed 1 h later at ZT3 or ZT15. Means ± SEM of immunoreactive (IR) cells are shown, *n* = 4 per group; * significant difference from corresponding control group, *p*<0.05.

Plasma CORT levels were significantly elevated in rats exposed to unpredictable restraint for 10 days and killed 1 h after stress onset on Day 10, compared to rats exposed to predictable restraint and control rats that were not exposed to stress (F_2,18_ = 19.86, *p*<0.0001; Fig. 10). CORT levels were higher in rats exposed to stress at night (ZT15) than in those exposed to stress in the day (ZT3) (F_1,18_ = 9.61, *p*<0.01; Fig. 10). The effect of repeated daily unpredictable and predictable restraint stress on PER1 and FOS expression on Day 10 is shown in Fig. 11, with statistical analyses in Table 5. No effects were observed in the SCN (Fig. 11A). Both unpredictable and predictable restraint stress led to significant increases in PER1 and FOS levels in the PVN at ZT3 (Fig. 11B), and in the DMH and Pi in rats exposed to stress at both ZT3 and ZT15 (Figs. 11C, D). Once again, in stark contrast PER1 and FOS expression in BNSTov and CEAl were significantly suppressed, but only following repeated unpredictable restraint stress and only at ZT3 (Figs. 11E, F).

**Table 5:** Statistical analysis of the effect of repeated daily predictable vs. unpredictable restraint stress and of time of day (ZT) on PER1 and FOS expression in limbic and hypothalamic brain regions.

Structure/Protein	Predictability	ZT (time of sacrifice)	Predictability × ZT
**SCN/**			
PER1	F_(2,18)_ = 1.03, ns	F_(1,18)_ = 379.79, *p*<0.0001	F_(2,18)_ = 0.86, ns
FOS	F_(2,18)_ = 1.37, ns	F_(1,18)_ = 97.48, *p*<0.0001	F_(2,18)_ = 0.44, ns
**PVN/**			
PER1	F_(2,17)_ = 10.81, *p*<0.001	F_(1,17)_ = 2.67, ns	F_(2,17)_ = 0.9, ns
FOS	F_(2,17)_ = 9.18, *p*<0.01	F_(1,17)_ = 3.44, ns	F_(2,17)_ = 1.99, ns
**DMH/**			
PER1	F_(2,18)_ = 8.81, *p*<0.01	F_(1,18)_ = 0.32, ns	F_(2,18)_ = 0.09, ns
FOS	F_(2,16)_ = 7.66, *p*<0.01	F_(1,16)_ = 4.45, ns	F_(2,16)_ = 3.46, ns
**Pi/**			
PER1	F_(2,18)_ = 40.76, *p*<0.0001	F_(1,18)_ = 2.72, ns	F_(2,18)_ = 3.25, ns
FOS	F_(1,17)_ = 28.17, *p*<0.0001	F_(2,17)_ = 0.12, ns	F_(2,17)_ = 0.75, ns
**BNSTov/**			
PER1	F_(2,18)_ = 4.86, *p*<0.05	F_(1,18)_ = 44.1, *p*<0.0001	F_(2,18)_ = 6.14, *p*<0.01
FOS	F_(2,18)_ = 4.25, *p*<0.05	F_(2,18)_ = 9.42, *p*<0.01	F_(2,18)_ = 4.92, *p*<0.05
**CEAl/**			
PER1	F_(2,18)_ = 2.11, ns	F_(1,18)_ = 48.51, *p*<0.0001	F_(2,18)_ = 5.53, *p*<0.05
FOS	F_(2,18)_ = 4.02, *p*<0.05	F_(1,18)_ = 1.24, ns	F_(2,18)_ = 5.22, *p*<0.05

ns, not significant.

## Discussion

Although there is growing evidence supporting the integration of the circadian and stress systems [1], [50], [53], little is known about the mechanisms that dictate how stressful events can affect circadian rhythmicity. In the present study, we examined the effects of stress on the expression of the circadian clock protein, PER1. Specifically, we assessed the impact of several stress variables that included the type of stress, time of exposure and mode of presentation of the stressor, on stress-induced changes in PER1 expression. Our results strongly allude to the notion that PER1 may be acting as an intermediary between the circadian and stress systems, by mediating the effect of stress on circadian molecular rhythms. Overall, our findings demonstrate that clock proteins in the brain are modifiable by stress, as previously suggested [4], [11], [12], [14]. Stress-induced disruptions in clock protein expression may consequently disrupt local oscillations within neural circuits that are important in regulating behavior and motivation.

A primary finding of this study is that categorically different stressors, namely processive versus systemic, have differential effects on the expression of PER1 and of FOS in two regions of the forebrain, the BNSTov and CEAl. Specifically, whereas systemic stressors increased the expression of PER1 and FOS in these regions, processive stressors suppressed their expression. In all other regions tested, including the PVN, DMH and Pi, both types of stressors increased PER1 and FOS levels. Additional data are required in order to determine whether the increase in PER1 levels are attributed to an increase in the number of cells recruited within the specific brain regions, or to an increase in the amount of protein expressed per cell. As expected, regardless of their different effects on PER1 and FOS expression, all stressors studied led to acute increases in plasma CORT levels.

Systemic stressors disrupt internal homeostasis and relay stress information directly to effector neurons in the hypothalamus [54], [55], whereas processive stressors first engage the central extended amygdala, in part composed of the BNSTov and CEAl, through higher order cortical processing and consequent activation of limbic-hypothalamic circuitry [56]. The present results show that PER1 and FOS expression in the central extended amygdala is differentially sensitive to qualitative differences between processive and systemic stressors, to which other regions of the forebrain do not respond. FOS expression in the forebrain is known to be strongly and rapidly modulated by stress [57], [58], and has been shown to be suppressed in the BNSTov and CEAl following exposure to processive stressors [59]. The functional significance of this processive stress-induced inhibition is not yet clear, but one current hypothesis is that GABAergic projections, which are found in abundance in nuclei of the central extended amygdala, are suppressed by processive stress, leading to increases in overall output from the amygdala and subsequent facilitation of autonomic and neuroendocrine responses [59].

A second finding is that, in general, the effects of nighttime stress on PER1 and FOS expression are similar though much attenuated, compared to the effects of daytime stress exposure. These data are consistent with several other studies that show that the effects of stress are dependent on the time of day [60]–[62]. The time-of-day effect of stress exposure on PER1 and FOS expression may be due to the diurnal variation in basal PER1 and FOS levels, or to daily changes in the sensitivity of the target structures to stress stimuli. Such a phase-dependent sensitivity exists in the SCN, whereby clock gene expression following light exposure is more robust during the night than day [63]–[65]. Similarly, adrenal sensitivity to adrenocorticotropic hormone in rodents is much higher during the subjective night, leading to elevated levels of basal CORT [49], [66]. Accordingly, we observed higher stress-induced increases in plasma CORT levels during the night.

In the present study, we found that daily predictable exposure to restraint for 10 days attenuated the PER1 and FOS responses to an acute restraint stress in the BNSTov and CEAl. This observation is in contrast to the lack of effect of unpredictable stress on the response of PER1 and FOS to acute stress in these two regions. In rats previously exposed to daily unpredictable stress, acute exposure to restraint suppressed PER1 and FOS expression in the BNSTov and CEAl and increased expression in the PVN, DMH and Pi, replicating the effects of acute restraint stress exposure seen in rats that were not pre-exposed to stress. The finding that the effect of restraint on PER1 and FOS in the BNSTov and CEAl habituates following repeated exposure to predictable, but not unpredictable, restraint stress emphasizes the ability of these nuclei to differentiate not only between qualitative differences in the type of stress, but also between the predictability of the stressor. However, whether the process of integrating stressor predictability takes place in the CEAl and BNSTov, or in upstream structures that project to the BNSTov and CEAl, remains to be determined. We also found that the SCN appeared to be immune to the effects of repeated daily acute stressors, although others have shown that clock gene expression in the SCN of mice can be affected by repeated stress [45], [67], [68]. Lastly, consistent with our observations of a habituation in plasma CORT secretion to repeated daily predictable restraint, there is ample evidence supporting habituation of HPA secretory responses to repeated daily predictable versus acute stress [69]–[71]. Repeated daily unpredictable stress has also been shown to produce enhanced basal glucocorticoid levels, with no indication of habituation over a long-term stress paradigm [72].

In the present study, all stressors administered consistently elevated circulating plasma CORT levels; this was mirrored by an increase in PER1 and FOS expression in all regions studied except the BNSTov and CEAl, where exposure to processive stressors suppressed PER1 and FOS expression despite the high levels of circulating CORT. Glucocorticoid hormones have been implicated in the regulation of clock gene rhythms in peripheral tissues and in the forebrain of rats. For example, we have shown that loss of endogenous glucocorticoids via adrenalectomy (ADX) or loss of glucocorticoid signaling via genetic deletion of brain GR blunt the rhythms of expression of PER2 in the BNSTov and CEAl [9], [11], [14], [73]. Blunted PER2 rhythms in ADX rats can be restored by exogenous CORT replacement via the drinking water, a regimen that mimics endogenous CORT rhythms [12]–[14]. A role for glucocorticoid signaling in daily PER2 rhythms is also supported by the presence of a GRE in the promoter region of *Per2*, as is the case with *Per1* [25], [74]. A direct role for glucocorticoid hormones in stress-induced increases of PER1 expression in the PVN, DMH and Pi remains to be demonstrated. Similarly, it is currently unknown whether stress-induced elevation in circulating glucocorticoids may also be involved in the suppressive effect of processive stressors on PER1 expression in the BNSTov and CEAl. However, inhibitory effects of elevated levels of glucocorticoids on a variety of peptides in the PVN have been previously reported [75].

Lastly, our data consistently highlight the identical trends in PER1 and FOS expression in response to different stressors, in the forebrain. However, it remains to be determined whether the same stress-induced signaling mechanisms that regulate *cFos* expression also regulate *Per1* in parallel. The similarity in the stress-induced behavior of both proteins supports the hypothesis that in addition to its role in the clock mechanism, *Per1* functions as an IEG [25], [76]. According to this, PER1 may serve as a transcription factor or effector protein capable of rapidly relaying stress information to circadian oscillators in the forebrain.

The functional significance of subordinate clock outputs in stress-responsive limbic and hypothalamic nuclei is largely unknown. It remains to be determined whether stress-induced changes in PER1 levels alter local rhythms of expression within these subordinate oscillators, uncoupling them from the SCN and consequently disrupting circadian outputs from these nuclei. Alternatively, the PER1 response to stress may be involved in systems external to the clock [20], [22], [26]–[28], [77], [78]. In conclusion, the data presented in the current study demonstrate that the mechanism that controls the expression of PER1 in the forebrain and hypothalamus is highly vulnerable to stress, and support the hypothesis that PER1 may integrate circadian and stress information by acting as an interface between both systems in the brain.
